# Dr. G. V. Black’s Conclusions Reviewed

**Published:** 1897-12

**Authors:** E. A. Bogue

**Affiliations:** New York


### DR. G. V. BLACK'S CONCLUSIONS REVIEWED.[1]

BY DR. E. A. BOGUE, NEW YORK.

In answer to your request for a communication that discusses the subject of Dr. Black's recent papers, I experience the same difficulty that I presume Dr. Black encountered at the beginning of his efforts to record the results of his experiments,—namely, the difficulty of pronouncing one's self.

I conceive that the more we discuss and work upon the facts which he has given, the clearer will become our concept of the truth as he meant to pronounce it.

To state only that "caries of the teeth is not dependent upon any condition of the tissues of the teeth, but on conditions of their environment," would seem to be stating something quite different from the statement in the *Digest:* "That the structure of the teeth is not a factor in their liability to caries *further than that pits*, *grooves*, *fissures*, and *roughness of surfaces* give opportunity by inviting lodgement and facilitating the growth of micro-organisms at particular points." So far as I am able to understand, the *condition* of the tissues of the teeth is involved in roughness or smoothness, in roundness or flatness, in density (specific gravity) or looseness of texture.

All these are conditions as well as the forms of the teeth.

In the *Dental Cosmos* for April, 1896, page 298, Dr. Black says that his work upon the teeth did not include variations in the hardness of the teeth to cutting instruments. This is a very important exception, and in view of it I am quite ready to concede all the rest of Dr. Black's findings in regard to the non-liability of the dentine of so-called soft teeth to decay, provided the surroundings, or, as he says, environments, are not promotive of that process.

But what, in few words and plain English, does Dr. Black mean by this? So far as I understand, he means that frail teeth (and I will say frail to the cutting instrument also), if wholly covered by the protective enamel, or, where that enamel is defective, if the defect is made good by accurately inserted fillings of indestructible material, that that tooth or those teeth may be indefinitely preserved in health and usefulness if kept clean. My clinical observations, accompanied by my written records carefully kept since I began practice, amply demonstrate the truth of this position.

---

[1] Read before The New York Institute of Stomatology, October 5, 1897.

Thus far I can follow Dr. Black's lead with great delight, but further than that I am not prepared to go. I find that my records teach me that the hard teeth that will strike fire with an instrument, as we all have doubtless seen at times, are invariably shorter, and consequently more rounded than those softer teeth (soft to the cutting instrument and of a chalky feel) that so often induce despair in the mind of the operator.

In a communication which I had the honor to make to the Société Odontologique de France in December, 1884, I stated as a result of long observation, "that if the crown of the human tooth is well formed it is more or less yellowish, short and rounded, more slender at the neck, and should only touch its neighbor at a single point, if the arrangement of the teeth in the mouth is what is called normal or ideal, and it will be noticed that this point is almost never attacked by caries; it is the region between this exact point of contact and the gum where proximal caries makes its first attacks.

"If, on the contrary, the tooth is frail and of a loose texture, it is more or less light in color, it is long instead of being short from the neck to the points of the cusps, narrow upon its antero-posterior diameter, broad from one side to the other laterally, and flattened. Teeth offering these characteristics are always more subject to caries, and very often they are irregularly placed in the mouth, which allows food to be crowded between the teeth, and often it cannot be removed." I should, perhaps, not have the hardihood to recall this communication were it not that my records fully bear out the statements there made in regard to a fairly large number of patients that I have been enabled to see from time to time during from ten to twenty-five years or more.

These statements were based upon the hypothesis mentioned above, that dense-looking and yellowish teeth, if only coated with enamel or with indestructible substitutes for the enamel where the enamel is defective, are less liable to caries than teeth of the opposite description.

And this for mechanical reasons,—namely, that their formation is such that they are less prone to catch and retain particles of food between or around them than teeth that are differently formed and frequently differently placed in the mouth.

It will be noticed that this in no way militates against the statements, taken altogether, made by Dr. Black, for he simply antagonizes the notion that what are called frail teeth are necessarily doomed to destruction.

I regret that Dr. Black's investigations do not yet lead him into the field of enamel, for while the enamel is intact the dentine does not decay.

I should expect to be confronted with the appearance well known to microscopic observers, of quite large decalcified spots in dentine where no perceptible opening exists in the enamel; my reply to that would be that I have not yet seen or heard of cases in which the enamel covering was properly formed, where anything more than interglobular spaces were found in the dentine, but I have several slides where beneath defective enamel quite large disintegrations exist in the dentine.

If clinical observations are to be admitted into this discussion, permit me to say that I have two lady patients in America and one in Russia whose teeth have been under my observation from fourteen to twenty-four years, the teeth of all of whom can be cut down about as a slate-pencil could, and yet no one of these ladies has ever lost a tooth, and only the one in Russia during her confinement had pain in her wisdom-tooth. Some of the fillings put in fourteen and more years ago for all these ladies are still in their places, and quite a large number have been in place more than ten years; two of these ladies are or have been the mothers of four or five children each. The Russian has teeth that are badly formed, flattened at the sides, and very difficult to cleanse, and yet they are all being preserved, and best of all are the gold fillings wherever I was able to use gold.

---

